# Osteopontin: a leading candidate adhesion molecule for implantation in pigs and sheep

**DOI:** 10.1186/2049-1891-5-56

**Published:** 2014-12-17

**Authors:** Greg A Johnson, Robert C Burghardt, Fuller W Bazer

**Affiliations:** Department of Veterinary Integrative Biosciences, Texas A&M University, College Station, TX 77843-4458 USA; Department of Animal Science, Texas A&M University, College Station, TX 77843 USA

**Keywords:** Implantation, Integrins, Psteoponti, Pigs, Sheep

## Abstract

Osteopontin (OPN; also known as Secreted Phosphoprotein 1, SPP1) is a secreted extra-cellular matrix (ECM) protein that binds to a variety of cell surface integrins to stimulate cell-cell and cell-ECM adhesion and communication. It is generally accepted that OPN interacts with apically expressed integrin receptors on the uterine luminal epithelium (LE) and conceptus trophectoderm to attach the conceptus to the uterus for implantation. Research conducted with pigs and sheep has significantly advanced understanding of the role(s) of OPN during implantation through exploitation of the prolonged peri-implantation period of pregnancy when elongating conceptuses are free within the uterine lumen requiring extensive paracrine signaling between conceptus and endometrium. This is followed by a protracted and incremental attachment cascade of trophectoderm to uterine LE during implantation, and development of a true epitheliochorial or synepitheliochorial placenta exhibited by pigs and sheep, respectively. In pigs, implanting conceptuses secrete estrogens which induce the synthesis and secretion of OPN in adjacent uterine LE. OPN then binds to αvβ6 integrin receptors on trophectoderm, and the αvβ3 integrin receptors on uterine LE to bridge conceptus attachment to uterine LE for implantation. In sheep, implanting conceptuses secrete interferon tau that prolongs the lifespan of CL. Progesterone released by CL then induces OPN synthesis and secretion from the endometrial GE into the uterine lumen where OPN binds integrins expressed on trophectoderm (αvβ3) and uterine LE (identity of specific integrins unknown) to adhere the conceptus to the uterus for implantation. OPN binding to the αvβ3 integrin receptor on ovine trophectoderm cells induces *in vitro* focal adhesion assembly, a prerequisite for adhesion and migration of trophectoderm, through activation of: 1) P70S6K via crosstalk between FRAP1/MTOR and MAPK pathways; 2) MTOR, PI3K, MAPK3/MAPK1 (Erk1/2) and MAPK14 (p38) signaling to stimulate trohectoderm cell migration; and 3) focal adhesion assembly and myosin II motor activity to induce migration of trophectoderm cells. Further large *in vivo* focal adhesions assemble at the uterine-placental interface of both pigs and sheep and identify the involvement of sizable mechanical forces at this interface during discrete periods of trophoblast migration, attachment and placentation in both species.

**Electronic supplementary material:**

The online version of this article (doi:10.1186/2049-1891-5-56) contains supplementary material, which is available to authorized users.

## Introduction

Domestic animal models for research are generally underappreciated[1]; however, pigs and sheep offer unique characteristics of pregnancy, as compared to rodent or primate models, and studies of pigs and sheep have provided significant insights into the physiology of implantation including: 1) elongation of the blastocyst into a filamentous conceptus; 2) the protracted peri-implantation period of pregnancy when the conceptus is free within the uterine lumen requiring extensive paracrine signaling between conceptus and endometrium, as well as nutritional support provided by uterine secretions; 3) a protracted and incremental attachment cascade of trophectoderm to endometrial epithelium during implantation; and (4) development of a true epitheliochorial or synepitheliochorial placenta, respectively, that utilizes extensive uterine and placental vasculatures for hematotrophic nutrition, and placental areolae for histotrophic support of the developing fetuses. Our understanding of the complex mechanistic events that underlie successful implantation and placentation across species has been and will likely continue to be advanced by studies of pigs and sheep as biomedical research models and to increase reproductive success in animal agriculture enterprises providing high quality protein for humans.

### Overview of the biology of osteopontin (OPN)

OPN is a secreted extracellular matrix (ECM) protein that binds to a variety of cell surface integrins and several CD44 variants[2–6]. Integrins are transmembrane glycoprotein receptors composed of non-covalently bound α and β subunits that promote cell-cell and cell-ECM adhesion, cause cytoskeletal reorganization to stabilize adhesion, and transduce signals through numerous signaling intermediates[7, 8]. Integrin-mediated adhesion is focused within a primary mechanotransduction unit of dynamic structure and composition known as a focal adhesion whose size, composition, cell signaling activity and adhesion strength are force-dependent[2, 9]. The intrinsic properties of the ECM in different niches and tissue-level compartments affect the composition and size of focal adhesions that, in turn, modulate cell behavior including gene expression, protein synthesis, secretion, adhesion, migration, proliferation, viability and/or apoptosis[10]. Integrins are dominant glycoproteins in many cell adhesion cascades, including well defined roles in leukocyte adhesion to the apical surface of polarized endothelium for extravasation of leukocytes from the vasculature into tissues[11]. A similar adhesion cascade involving interactions between the ECM and apically expressed integrin receptors on the uterine luminal epithelium (LE) and conceptus (embryo and placental membranes) trophectoderm is proposed as a mechanism for attachment of the conceptus to the uterus for implantation; the initial step for the extensive tissue remodeling that occurs during placentation[12]. OPN is a leading candidate adhesion molecule for implantation in pigs and sheep[13].

OPN is an acidic member of the small integrin-binding ligand *N*-linked glycoprotein (SIBLING) family of proteins[9]. The breadth of literature pertaining to the diverse functions of OPN is extensive, and OPN has been independently identified as a protein associated with metastatic cancers (2ar), as an ECM protein of bones and teeth (OPN, BSP1, BNSP,SPP1), as a cytokine produced by activated lymphocytes and macrophages (early T-cell activation factor 1, Eta-1), and as a major constituent of the uterus and placenta during pregnancy[13–17]. In general, OPN is a monomer ranging in length from 264 to 301 amino acids. OPN contains a hydrophobic leader sequence characteristic of a secreted protein, a calcium phosphate apatite binding region of consecutive asparagine residues, a GRGDS sequence that interacts with integrins, a thrombin cleavage site, and two glutamine residues that are recognized substrates for transglutaminase-supported multimer formation[3, 5, 6]. Genes encoding OPN from different species present only moderate sequence conservation, except in the NH_2_-terminal region, around the Arg-Gly-Asp (RGD) integrin-binding sequence, and in the COOH-terminus[3, 5, 6, 18]. OPN undergoes extensive posttranslational modifications that can alter its function in different physiological microenvironments. These modifications include proteolytic cleavage, phosphorylation, glycosylation, sulfation and cross-linking with self and other macromolecules[19–23]. OPN is present on epithelial cells and in secretions of the gastrointestinal tract (including the liver), respiratory tract, kidneys, thyroid, breast, testes, uterus and placenta[24–32]. Other cell types that express OPN include leukocytes, smooth muscle cells, and highly metastatic cancer cells[33–35]. OPN is a multifunctional ECM protein reported to 1) stimulate cell-cell adhesion, 2) increase cell-ECM communication, 3) promote cell migration, 4) decrease cell death, 5) stimulate immunoglobulin production, 6) induce changes in phosphorylation of focal adhesion kinase and paxillin, 7) stimulate phosphotidylinositol 3′-kinase activity, 8) alter intracellular calcium levels, and 9) promote calcium phosphate deposition[36–42].

### Timeline of key advancements in understanding the role of OPN as an attachment factor for implantation

OPN was first observed in endometrial tissue when, in 1988, Nomura et al.,[43] performed *in situ* hybridization to localize OPN in mouse embryos, the endometrium from the gravid and non-gravid uterine horns of pregnant mice, and the endometrium from mice exposed to intrauterine injection of oil to induce a deciduoma. High levels of OPN mRNA were detected in the LE, but not GE, of the gravid uterine horns. Interestingly, epithelial expression of OPN appeared to be specific to pregnancy because little to no OPN mRNA was observed in the uterine LE of non-gravid or pseudopregnant mice[43]. In addition to the LE, high levels of OPN mRNA were localized to the granulated metrial gland (GMC) cells of decidual and deciduoma tissues, with lower numbers of OPN positive cells in the deciduoma of uteri[43]. It is noteworthy that these investigators were the first to argue that OPN plays a wider role than had previously been assumed, and that its functions are not confined to bone development. The decidual cells that express OPN have since been confirmed to be uterine natural killer (uNK) cells[44, 45]. Similar to expression in mice, immunocytochemical studies performed by Young and colleagues in 1990[25] localized OPN protein to the decidua of women; however, in contrast to mice, OPN was also expressed by the secretory phase endometrial GE. It was suggested that the absence of OPN in GE during the proliferative phase of the menstrual cycle indicated that changes in expression in GE of normal cycling endometrium were the result of hormonal regulation and that the function(s) of OPN in the endometrium might be associated with its ability to enhance cell attachment[25].

A significant conceptual advance regarding the function(s) of epithelia-derived OPN was made by Brown and co-workers[26] in 1992, when OPN mRNA and protein were localized to epithelial cells of a variety of organs, including the hypersecretory endometrial GE associated with pregnancy in women. In the secretory epithelia of all organs examined, OPN protein was associated with the apical domain of the cells, and when the luminal contents were preserved in tissue sections, proteins secreted into the lumen were positive for OPN staining. It was hypothesized that OPN secreted by epithelia, including uterine epithelia, binds integrins on luminal surfaces to effect communication between the surface epithelium and the external environment[26]. Between 1992 and 1996, Lessey and co-workers established that transient uterine expression of αvβ3 and α4β1 integrins defines the window of implantation in women[46–48] and that altered expression of these integrins correlates with human infertility[49, 50]. Noting that the αvβ3 and α4β1 integrin heterodimers present during the implantation window bind OPN, these investigators suggested involvement of OPN and integrins in trophoblast-endometrial interactions during the initial attachment phase of implantation[46].

Comprehensive examination of the temporal and spatial expression and hormonal regulation of uterine OPN mRNA and protein and integrin subunit proteins in the uteri and placentae of sheep (discussed in detail later in this review), performed from 1999 through 2002, provided the first strong evidence that OPN is a progesterone-induced secretory product of endometrial glands (histotroph) that binds integrins on apical surfaces of endometrial LE and conceptus trophectoderm to mediate attachment of uterus to trophectoderm for implantation[18, 29, 51, 52]. Indeed, pregnant Day 14 ewes, which lack uterine glands (uterine gland knockout, UGKO phenotype), exhibit an absence of OPN in uterine flushings compared with normal ewes, and do not maintain pregnancy through the peri-implantation period[53]. Similarly, functional intrauterine blockade of αv and β3 integrin subunits, that combine to form a major receptor for OPN, reduces the number of implantation sites in mice and rabbits[54, 55]. Further evidence for regulation of uterine OPN by sex steroids was provided by results from studies using human and rabbit models. Progesterone treatment increased OPN expression by human endometrial adenocarcinoma Ishakawa cells (*in vitro* findings, 2001) as well as endometrium of rabbits (*in vivo* findings, 2003)[56, 57]. In contrast, i.m. injection of estrogen induced expression of OPN in the uterine LE of cyclic pigs (*in vivo*, 2005)[58]. Results from pigs were the first to suggest that conceptuses can directly regulate the regional expression of OPN in the endometrium at specific sites of implantation through secretion of estrogens[58, 59]. Microarray studies from 2002 and 2005 strongly support a role for OPN during implantation[60–62]. Two reports confirmed that OPN is the most highly up-regulated ECM-adhesion molecule in the human uterus as it becomes receptive to implantation[60–62].

Research regarding OPN has begun to focus on its interactions with integrin receptors in the female reproductive tract. In 2009, Burghardt and colleagues[63] reported the *in vivo* assembly of large focal adhesions containing aggregates of αv, α4, α5, β1, β5, alpha actinin, and focal adhesion kinase (FAK) at the uterine-placental interface of sheep, that expand as pregnancy progresses. It is noteworthy that OPN was present along the surfaces of both uterine LE and trophectoderm, although it was not determined whether it co-localized to the focal adhesions[63]. Similar focal adhesions form during implantation in pigs[64, 65]. Affinity chromatography and immunoprecipitation experiments revealed direct *in vitro* binding of porcine trophectoderm αvβ6 and uterine epithelial cell αvβ3, and ovine trophectoderm αvβ6 integrins to OPN[64, 66]. These were the first functional demonstrations that OPN directly binds specific integrins to promote trophectoderm cell migration and attachment to uterine LE that may be critical to conceptus elongation and implantation. Recently (2014), Aplin and co-workers[67] employed three *in vitro* models of early implantation with Ishakawa cells to demonstrate that OPN potentially interacts with the αvβ3 integrin receptor during implantation in humans.

### Key events during the peri-implantation period of pigs and sheep

Communication and reciprocal responses between the conceptus and uterus are essential for conceptus survival during the peri-implantation period of pregnancy. These interactions also lay the critical physiological and anatomical groundwork for subsequent development of functional uterine LE, GE, stroma and placentae required to maintain growth and development of the conceptus throughout pregnancy. In a progesterone dominated uterine environment, establishment and maintenance of pregnancy in pigs and sheep requires; (i) secretion of estrogens or interferon tau, respectively, from the conceptus to signal pregnancy recognition[68–71], (ii) secretions from uterine LE and GE, i.e., histotroph, to support attachment, development and growth of the conceptus[72–74], and (iii) cellular remodeling at the uterine LE-conceptus trophectoderm interface to allow for attachment during implantation[8, 75, 76]. These events are orchestrated through endocrine, paracrine, autocrine and juxtracrine communication between the conceptus and uterus, and the complexity of these events likely underlies the high rates of conceptus mortality during the peri-implantation period of pregnancy[77, 78].

Implantation and placentation are critical events in pregnancy. Implantation failure during the first three weeks of pregnancy is a major cause of infertility in all mammals[77–80]. The process of implantation is highly synchronized, requiring reciprocal secretory and physical interactions between a developmentally competent conceptus and the uterine endometrium during a restricted period of the uterine cycle termed the “window of receptivity”. These initial interactions between apical surfaces of uterine LE and conceptus trophectoderm begin with sequential phases i.e., non-adhesive or pre-contact, apposition, and adhesion, and conclude with formation of a placenta that supports fetal-placental development throughout pregnancy[81–83]. Conceptus attachment first requires loss of anti-adhesive molecules in the glycocalyx of uterine LE, comprised largely of mucins that sterically inhibit attachment[52, 84, 85]. This results in “unmasking” of molecules, including selectins and galectins, which contribute to initial attachment of conceptus trophectoderm to uterine LE[86–88]. These low affinity contacts are then replaced by a repertoire of adhesive interactions between integrins and maternal ECM which appear to be the dominant contributors to stable adhesion at implantation[1, 8, 52, 89–91]. OPN is expressed abundantly within the conceptus-maternal environment in numerous species, including pigs and sheep[17, 29, 57, 59, 62, 92, 93].

### Osteopontin is structurally and functionally suited to support implantation of pig and sheep conceptuses

Depending on cell context and species, OPN expression can be regulated by multiple hormones and cytokines, including the sex steroids progesterone and estrogen[28, 51, 56–58, 94–98]. OPN mediates multiple cellular processes, such as cell-mediated immune responses, inflammation, angiogenesis, cell survival, and tumor metastasis primarily through integrin signaling[3, 5, 17, 99, 100]. Integrins are transmembrane glycoprotein receptors composed of non-covalently bound α and β subunits that participate in cell-cell and cell-ECM adhesion, cause cytoskeletal reorganization to stabilize adhesion, and transduce signals through numerous signaling intermediates[7, 8]. OPN has an expansive integrin receptor repertoire that includes RGD-mediated binding to αvβ3[101, 102], αvβ1[103], αvβ5[103], and α8β1[104], as well as alternative binding sequence-mediated interactions with α4β1[105], and α9β1[106]. OPN binding to these various receptors results in diverse effects including: (1) leukocyte, smooth muscle cell and endothelial cell chemotaxis; (2) endothelial and epithelial cell survival; and (3) fibroblast, macrophage and tumor cell migration[64, 66, 103, 104, 107]. Clearly, the ability to bind multiple integrin receptors to produce different cellular outcomes greatly increases OPN’s potential role(s) during conceptus development and implantation. Importantly, OPN contains a serine protease cleavage site that when activated generates bioactive OPN fragments[23, 108], and two glutamines that support multimerization of the protein[22]. It is notable that OPN is flexible in solution, allowing for simultaneous binding to more than one integrin receptor[16, 109]. Further, OPN can also exist in a polymerized form cross-linked by transglutaminase. Homotypic OPN bonds have high tensile strength, suggesting that self-assembly is involved in cell-cell and cell-matrix interactions[22]. These multimeric complexes may present multiple RGD sequences for simultaneous binding to integrins on multiple surfaces[22, 110]. Therefore, OPN has the potential to bind multiple proteins and to participate in assembly of multi-protein complexes that bridge and form the interface between conceptus to uterus during implantation.

### OPN expression, regulation and function in the uterus and placenta of gilts

A hallmark of pregnancy in pigs is the protracted peri-implantation period of pregnancy when conceptuses are free within the uterine lumen to elongate from spherical blastocysts to conceptuses with a filamentous morphology (Reviewed in[111]). Pig embryos move from the oviduct into the uterus about 60 to 72 h after onset of estrus, reach the blastocyst stage by Day 5, then shed the zona pellucida and expand to 2–6 mm in diameter by Day 10. At this stage, development of pig embryos diverges from that of rodents or primates. Within a few hours the presumptive placental membranes (trophectoderm and extra-embryonic endoderm) elongate at a rate of 30–45 mm/h from a 10 mm blastocyst to a 150–200 mm long filamentous form, after which further elongation occurs until conceptuses are 800–1,000 mm in length by Day 16 of pregnancy[111]. During this period of rapid elongation, porcine conceptuses secrete estrogen beginning on Days 11 and 12 to signal initiation of pregnancy to the uterus, and by Day 13 begin an extended period of incremental attachment to the uterine LE[17, 69]. The attached trophectoderm/chorion-endometrial epithelial bilayer develops microscopic folds, beginning about Day 35 of gestation, and these folds increase the surface area of contact between maternal and fetal capillaries to maximize maternal-to-fetal exchange of nutrients and gases[112].

In pigs, OPN is an excellent candidate for influencing this complex environment of pregnancy, because the OPN gene is located on chromosome 8 under a quantitative trait loci (QTL) peak for prenatal survival and litter size,[113]. The temporal and spatial expression of OPN in the porcine uterus and placenta is complex, with independent and overlapping expression by multiple cell types. Between Days 5 and 9 of the estrous cycle and pregnancy, OPN transcripts are detectable in a small percentage of cells in the sub-epithelial stratum compactum of the endometrial stroma[59]. The morphology and distribution of OPN mRNA- and protein-positive cells in the stratum compactum of the stroma on Day 9 of the estrous cycle and pregnancy suggest that these are immune cells. Certainly Eta-1/OPN, is an established component of the immune system that is secreted by activated T lymphocytes[15]. It is reasonable to speculate that because insemination in pigs is intrauterine, OPN expressing immune cells may protect against pathogens introduced during mating. A similar pattern of distribution of OPN-producing cells is also evident in the allantois of the placenta beginning between Days 20 and 25 of pregnancy, and the number of these cells increases as gestation progresses[58]. The identity of these cells remains to be determined.

**Figure 1:**
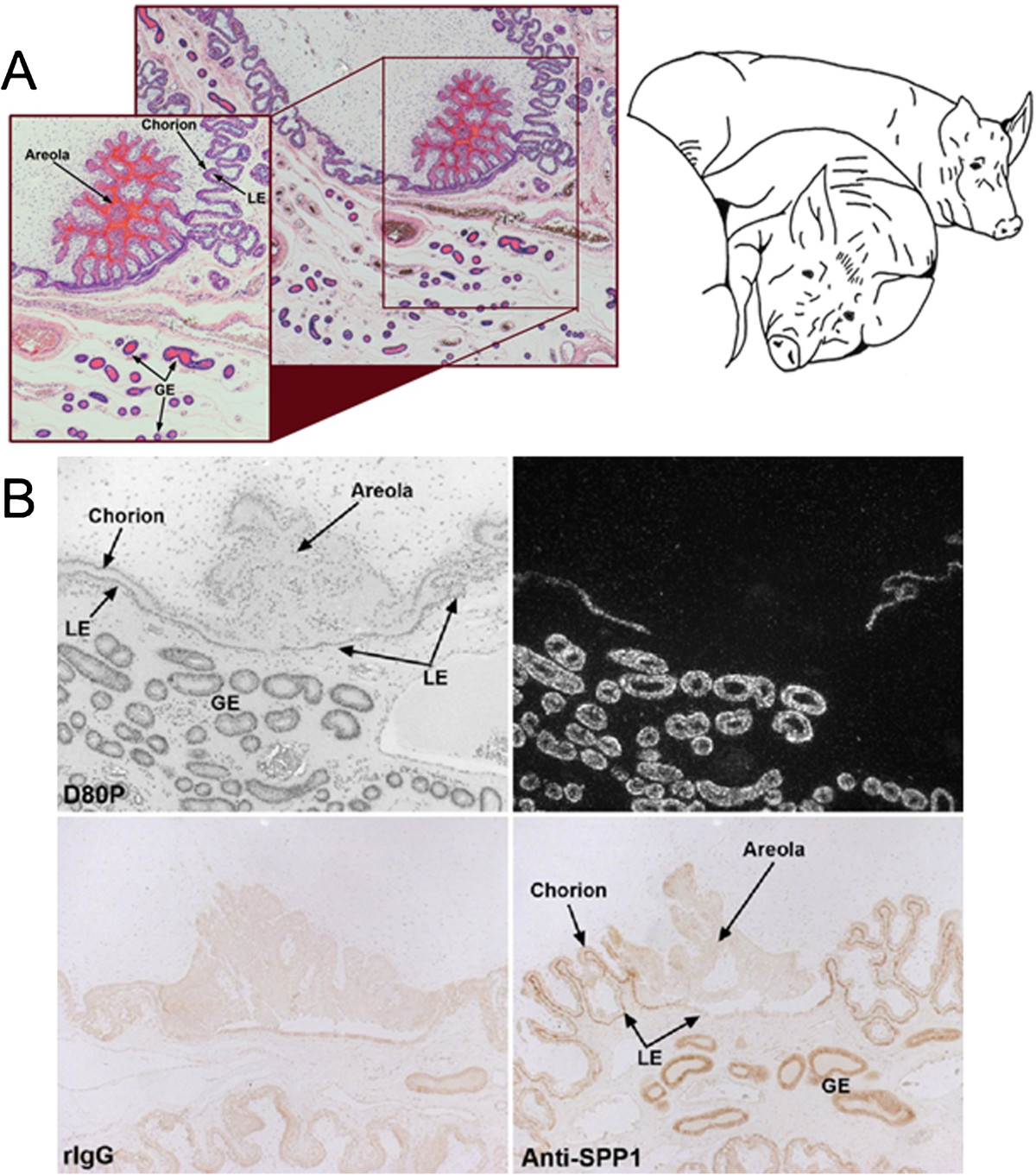
**OPN is synthesized and secreted from the luminal epithelium (LE) only at sites of direct attachment of uterus to placenta. A)** H&E stained paraffin embedded thin section of the uterine/placental interface of a Day 80 pregnant gilt illustrating an areola containing histotroph (note the intense red eosin protein staining) secreted by the glandular epithelium (GE). **B)** OPN mRNA (top panels) and protein (bottom panels) is expressed in the uterus of a Day 80 pregnant gilt (expression begins in luminal epithelium (LE) on Day 13, in GE by Day 35, and then in both cell types to term). Note that OPN is not detectable in uterine LE associated with areolae where there is no direct attachment of uterine LE to placental trophectoderm/chorion). This precise spatial distribution for OPN expression strongly suggests that it plays a role for attaching uterus to placenta during epitheliochorial placentation.

OPN expression in uterine LE increases markedly during the peri-implantation period of pigs, but is never observed in uterine LE during the estrous cycle[59]. OPN mRNA is initially induced by conceptus estrogens in discrete regions of the LE juxtaposed to the conceptus just prior to implantation on Day 13, then expands to the entire LE by Day 20 when firm adhesion of conceptus trophectoderm to uterine LE occurs[58]. However, OPN mRNA is not present in pig conceptuses[58, 59]. In contrast to mRNA, OPN protein is abundant along the apical surfaces of LE and trophectoderm/chorion, but only in areas of direct contact between the uterus and conceptus[58, 59]. Remarkably, OPN mRNA and protein are not present in uterine LE and chorion of areolae where the chorion does not attach to LE, but rather forms a “pocket” of columnar epithelial cells that take up and transport secretions of uterine GE into the placental vasculature by fluid phase pinocytosis[114] (Figure 1). OPN levels remain high at this interface throughout pregnancy[59], as do multiple integrin subunits that potentially form heterodimeric receptors that bind OPN[8, 84, 90].

All experimental and surgical procedures were in compliance with the Guide for Care and Use of Agricultural Animals in Teaching and Research and approved by the Institutional Animal Care and Use Committee of Texas A&M University.

**Figure 2:**
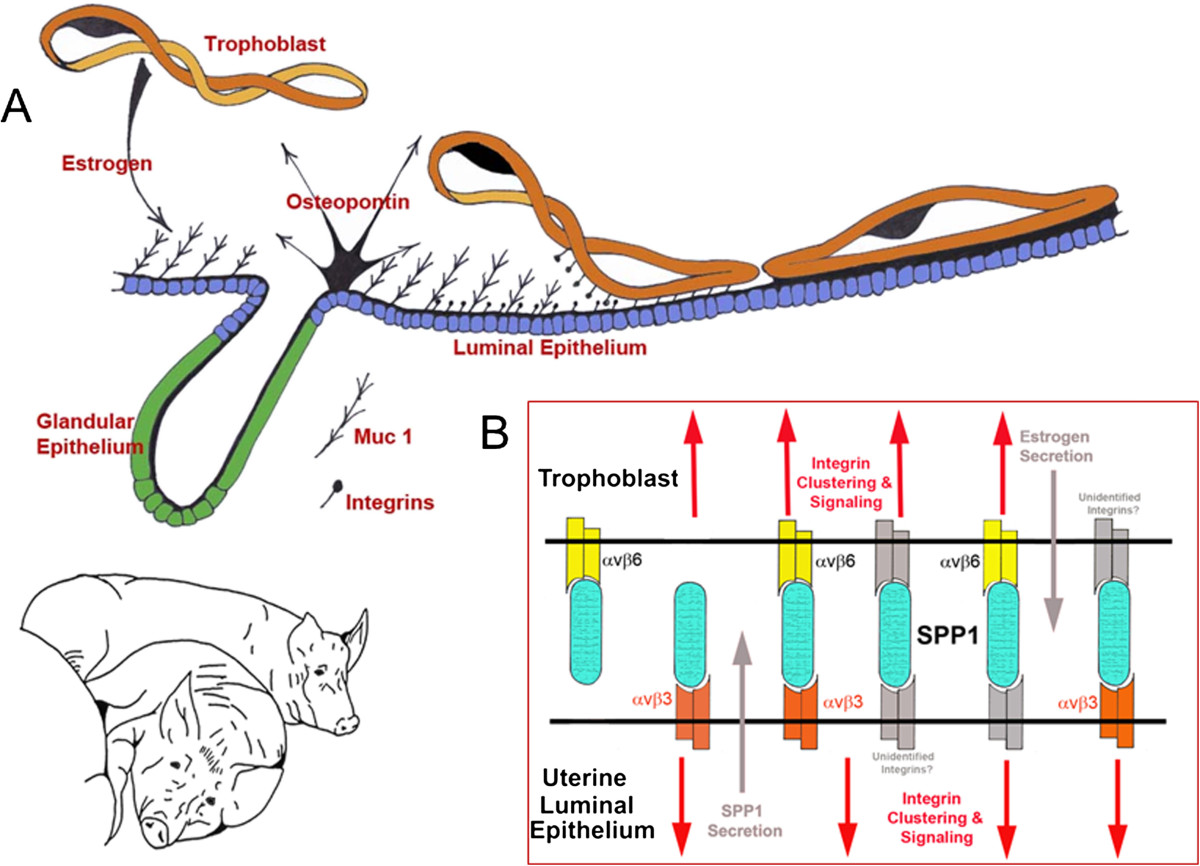
**Expression, regulation and proposed function of OPN produced by the uterine LE of pregnant pigs. A)** As porcine conceptuses (Trophoblast) elongate they secrete estrogens for pregnancy recognition. These estrogens also induce the synthesis and secretion of OPN (osteopontin) from the uterine LE (luminal epithelium) directly adjacent to the conceptus undergoing implantation[58]. The implantation cascade is initiated when progesterone from CL down-regulates Muc 1 on the surface of uterine LE[84]. This exposes integrins on the LE and trophoblast surfaces[84] for interaction with OPN, and likely other ECM proteins, to mediate adhesion of trophoblast to LE for implantation[58, 59, 64]. **B)** *In vitro* experiments have identified the αvβ6 integrin receptor on trophoblast, and the αvβ3 integrin receptor on LE as binding partners for OPN[64]. OPN may bind individually to these receptors to act as a bridging ligand between these receptors. Alternatively, OPN may serve as a bridging ligand between one of these receptors and an as yet unidentified integrin receptor expressed on the opposing tissue.

Affinity chromatography and immunoprecipitation experiments were performed to test whether the integrin subunits αv, α4, α5, β1, β3, β5, and β6, expressed by porcine trophectoderm cells (pTr2) and porcine uterine epithelial (pUE) cells, directly bind OPN. Detergent extracts of surface-biotinylated pig trophectoderm (pTr2) and uterine epithelial (pUE) cells were incubated with OPN-Sepharose and the proteins that bound to OPN were eluted with EDTA to chelate cations and release bound integrins. To identify these integrins, immunoprecipitation assays were performed using antibodies that successfully immunoprecipitated integrin subunits from pTr2 or pUE cell lysates. OPN directly bound the αvβ6 integrin heterodimer on pTr2 cells and αvβ3 on ULE cells[64]. OPN binding promoted dose- and integrin-dependent attachment of pTr2 and pUE cells, and stimulated haptotactic pTr2 cell migration, meaning that cells migrated directionally along a physical gradient of nonsoluble OPN[64]. Further, immunofluorescence staining revealed that both OPN and αv integrin subunit localized to the apical surface of cells at the interface between uterine LE and conceptus trophectoderm at Day 20 of pregnancy. The αv integrin subunit staining pattern revealed large aggregates at the junction between trophectoderm and uterine LE, suggesting the formation of OPN-induced *in vivo* focal adhesions at the apical surfaces of both conceptus trophectoderm and uterine LE that facilitate conceptus attachment to the uterus for implantation. The β3 subunit appeared in aggregates on the apical surface of LE cells, but not trophectoderm cells, fitting with affinity chromatography data indicating direct binding of αvβ3 on pUE cells to OPN[64]. Finally, OPN-coated microspheres revealed co-localization of the αv integrin subunit and talin to focal adhesions at the apical domain of pTr2 cells *in vitro*[64]. Collectively, results support that OPN binds integrins to stimulate integrin-mediated focal adhesion assembly, attachment, and cytoskeletal force-driven migration of pTr2 cells to promote conceptus implantation in pigs (Figure 2).

In addition to expression in LE during the peri-implantation period, total uterine OPN mRNA increases 20-fold between Days 25 and 85 of gestation due to induction of OPN expression in uterine GE[59]. The initial significant increase in GE is delayed until between Days 30 and 35 when placental growth and placentation are key events in pregnancy in pigs[5]. OPN expression in GE during later stages of pregnancy is also observed sheep[115], and a microarray study in rats showed that OPN expression increased 60-fold between Day 0 of the estrous cycle and Day 20 of pregnancy, likely within the decidua[116]. Indeed, OPN is expressed by uterine natural killer (uNK) cells of the mouse decidua[44, 45]. Secretions of GE in livestock, and the secretions of decidua in rodents and primates, are critical to support implantation, placentation, and fetal growth and development[117, 118]. OPN is also expressed in uterine GE of Day 90 of pseudopregnant pigs, suggesting that maintenance of secretion of progesterone by CL is responsible for expression of OPN in GE[58]. Progesterone also regulates OPN expression in the GE of sheep and rabbits[51, 54], as well as OPN synthesis by human Ishikawa cells[56].

However, the involvement of progesterone in the regulation of OPN in uterine GE is complex as indicated by recent analysis of long-term progesterone treatment on the expression of OPN in pigs in the absence of ovarian or conceptus factors. In addition to OPN expression, other established progesterone targets including progesterone receptor (PGR) as an index of progesterone’s ability to negatively regulate GE gene expression[119], acid phosphate 5, tartrate resistant (ACP5, commonly referred to as uteroferrin) as an index of progesterone’s ability to positively regulate early pregnancy GE gene expression[120], and fibroblast growth factor 7 (FGF7, commonly referred to as keratinocyte growth factor) provide an index of progesterone’s ability to positively regulate gene expression in uterine GE beyond the peri-implantation period[121]. Pigs were ovariectomized on Day 12 of the estrous cycle when progesterone secretion from CL is high and treated daily with intramuscular injections of progesterone or vehicle for 28 days[122, 123]. As anticipated, PGR mRNA decreased, uteroferrin mRNA increased, and FGF7 mRNA increased in uterine GE of pigs injected with progesterone[123]. Surprisingly, long-term progesterone, in the absence of ovarian and/or conceptus factors, did not induce OPN expression in uterine GE[123]. It is currently hypothesized that the hormonal milieu necessary for the production of individual components of histotroph varies, and may require specific servomechanisms, similar to those for sheep and rabbits, which involve sequential exposure of the pregnant uterus to ovarian, conceptus, and/or uterine factors that include progesterone, estrogens and IFNs[124–126]. Recently OPN expression was compared in placental and uterine tissues supplying a normally sized and the smallest fetus carried by hyperprolific Large White and Meishan gilts. Not only were levels of OPN strikingly different between the two breeds of pigs, but OPN was higher in the LE and GE of uteri surrounding smaller sized fetuses, suggesting OPN may be associated with placental efficiency[127].

### OPN expression, regulation and function in the uterus and placenta of ewes

Similar to pigs, the conceptuses of sheep remain free-floating within the uterine lumen as they elongate from spherical blastocysts to conceptuses with a filamentous morphology (Reviewed in[88]). Sheep embryos enter the uterus on Day 3, develop to spherical blastocysts and then, after hatching from the zona pellucida, transform from spherical to tubular and filamentous conceptuses between Days 12 and 15 of pregnancy, with extra-embryonic membranes extending into the contralateral uterine horn between Days 16 and 20. During this period of rapid elongation, the mononuclear trophoblast cells of ovine conceptuses secrete interferon tau between Days 10 and 21 of pregnancy, and implantation begins on Day 16 as trophectoderm attaches to the uterine LE[70, 88]. The ovine placenta eventually organizes into discrete regions called placentomes that are composed of highly branched placental chorioallantoic villi termed cotyledons which grow rapidly and interdigitate with maternal aglandular endometrial crypts termed caruncles. Approximately 90% of the blood from the uterine artery flows into the placentomes for nutrient transfer from the maternal uterine circulation to the fetus and exchange of gasses between these tissue compartments[128].

The temporal and spatial expression of OPN in the uteri and placentae of sheep is similar to that previously described for the pig, except: 1) unlike in the pig, OPN is not expressed by uterine LE; 2) induction of OPN in uterine GE occurs earlier than in the pig during the peri-implantation period, and expression in the GE is regulated by progesterone; 3) OPN is a prominent component of the stratum compactum stroma; and 4) although large focal adhesions assemble during the peri-implantation period of pigs, they are not observed at the uterine-placental interface until later stages of pregnancy in sheep.

OPN mRNA and protein are present in a small population of cells scattered within the stratum compactum stroma immediately beneath the endometrial LE during the early stages of the estrous cycle and pregnancy in sheep[18]. OPN-producing cells are also present in the allantois of the ovine placenta beginning between Days 20 and 25 of pregnancy and increase in number as gestation progresses[17]. As hypothesized for pigs, these are presumed to be immune cells because Eta-1/OPN is a prominent player in the immune system[15]. In contrast to pigs, in which the OPN-expressing endometrial cells are readily evident in the stratum compactum stroma throughout pregnancy, these cells are difficult to discern in the sheep due to an increase in expression of OPN by stromal cells between Days 20 and 25 gestation[129]. In pregnant mice and primates, OPN in decidualized stroma is considered to be a gene marker for decidualization[130, 131]. Decidualization involves transformation of spindle-like fibroblasts into polygonal epithelial-like cells that are hypothesized to limit conceptus trophoblast invasion through the uterine wall during invasive implantation[118]. Although Mossman[132] and Kellas[133] described decidual cell characteristics in the placentomal crypts of sheep and antelope, their reports were largely ignored, and decidualization was not thought to occur in species with central and noninvasive implantation characteristic of domestic animals. However, endometrial stromal cells do increase in size and become polyhedral in shape in pregnant ewes following conceptus attachment, and the classical decidualization markers desmin and α-smooth muscle actin are expressed in these cells, suggesting that OPN expression in this stromal compartment is part of a uterine decidualization-like response to the conceptus during ovine pregnancy[129]. In contrast, no morphological changes in uterine stroma, nor induction of OPN mRNA and protein, or desmin protein, were detected during porcine pregnancy[129]. One of the primary roles of decidua in invasive implanting species is to restrain conceptus trophoblast invasion to a circumscribed region of the endometrium. Both pigs and sheep have noninvasive implantation, but the extent of conceptus invasion into the endometrium differs between these two species. Pig conceptuses undergo true epitheliochorial placentation in which uterine LE remains morphologically intact throughout pregnancy and the conceptus trophectoderm simply attaches to the apical surface of uterine LE surface without contacting uterine stromal cells[134]. Synepitheliochorial placentation in sheep involves extensive erosion of the LE due to formation of syncytia with binucleate cells of the trophectoderm. After Day 19 of pregnancy, conceptus tissue is opposed to, but does not penetrate ovine uterine stroma[135]. Although speculative, differences in stromal expression of OPN between these species suggest that the extent of decidualization is correlated positively with degree of conceptus invasiveness.

In contrast to pigs, OPN is not synthesized by sheep uterine LE, but is nonetheless a component of histotroph secreted from the endometrial GE into the uterine lumen of pregnant ewes as early as Day13. It is not secreted by uterine GE of cyclic ewes[18, 29]. OPN mRNA is detected in some uterine glands by Day 13, and is present in all glands by Day 19 of gestation[18]. Progesterone induces expression of OPN in the endometrial GE, and induction is associated with a loss of PGR in uterine GE. Analysis of uterine flushings from pregnant ewes has identified a 45 kDa fragment of OPN with greater binding affinity for αvβ3 integrin receptor than native 70 kDa[29, 51, 52, 108]. Comparison of the spatial distribution of OPN mRNA and protein by *in situ* hybridization and immunofluorescence analyses of cyclic and pregnant ovine uterine sections has provided significant insight into the physiology of uterine OPN during pregnancy. OPN mRNA increases in the endometrial GE during the peri-implantation period; however, it is not present in LE or conceptus trophectoderm[18]. In contrast, immunoreactive OPN protein is present at the apical surfaces of endometrial LE and GE, and on trophectoderm where the integrin subunits αv, α4, α5, β1, β3, and β5 are expressed constitutively on the apical surfaces of trophectoderm and endometrial LE and could potentially assemble into several heterodimers that could serve as receptors for OPN including αvβ3, αvβ1, αvβ5, α4β1, and α5β1 heterodimers which[29, 52]. These results strongly suggest that OPN is a component of histotroph secreted from GE into the uterine lumen of pregnant ewes in response to progesterone, and that OPN binds integrin receptors expressed on endometrial LE and conceptus trophectoderm.

**Figure 3:**
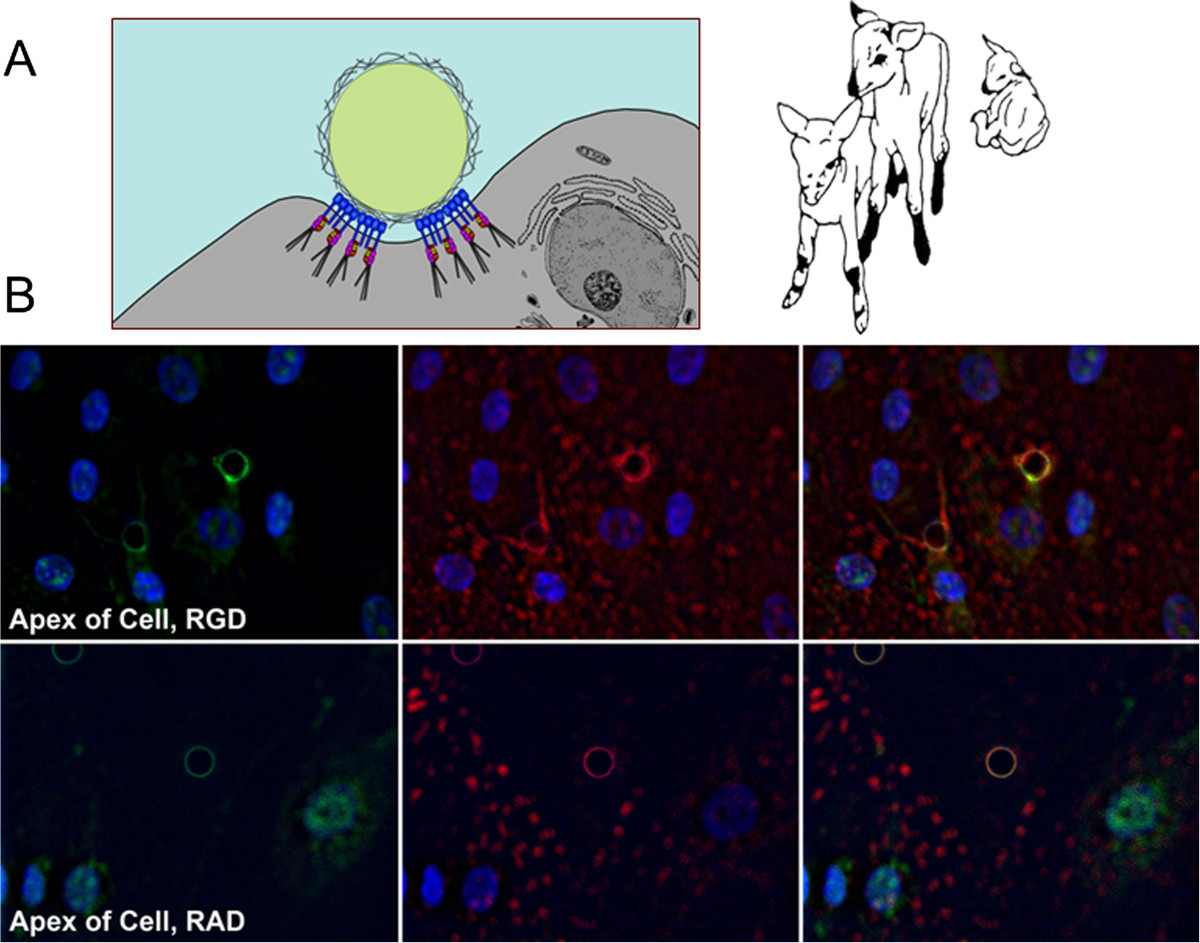
**OPN stimulates** ***in vitro*** **activation of integrin receptors to form focal adhesions at the apical surface of oTr1 cells. A)** Cartoon illustrating a polystyrene bead coated with recombinant rat OPN containing an intact RGD integrin binding sequence, and allowed to settle onto a cultured oTr1 cell. Note the illustrated representation of aggregated integrins, indicative of focal adhesion assembly, at the interface between the surface of the bead and the apical membrane of the cell[52, 64, 66]. **B)** Immunofluorescence co-localization (left panels) to detect the aggregation of αv integrin subunit (right panels) and talin middle panels), an intracellular component of focal adhesions, around beads coated with recombinant rat OPN containing an intact RGD integrin binding sequence (RGD) or coated with recombinant OPN containing a mutated RAD sequence that does not bind integrins[66]. Optical slice images from the apical plasma membrane of oTr1 cells are shown. Note the apical focal adhesions represented by immunofluo rescence co-localization (yellow color) of the integrin αv subunit with talin that results from integrin activation in response to binding of intact OPN on the surface of the bead. No apical focal adhesions were induced by beads coated with mutated OPN as evidenced by lack of integrin αv and talin aggregation around the bead.

**Figure 4:**
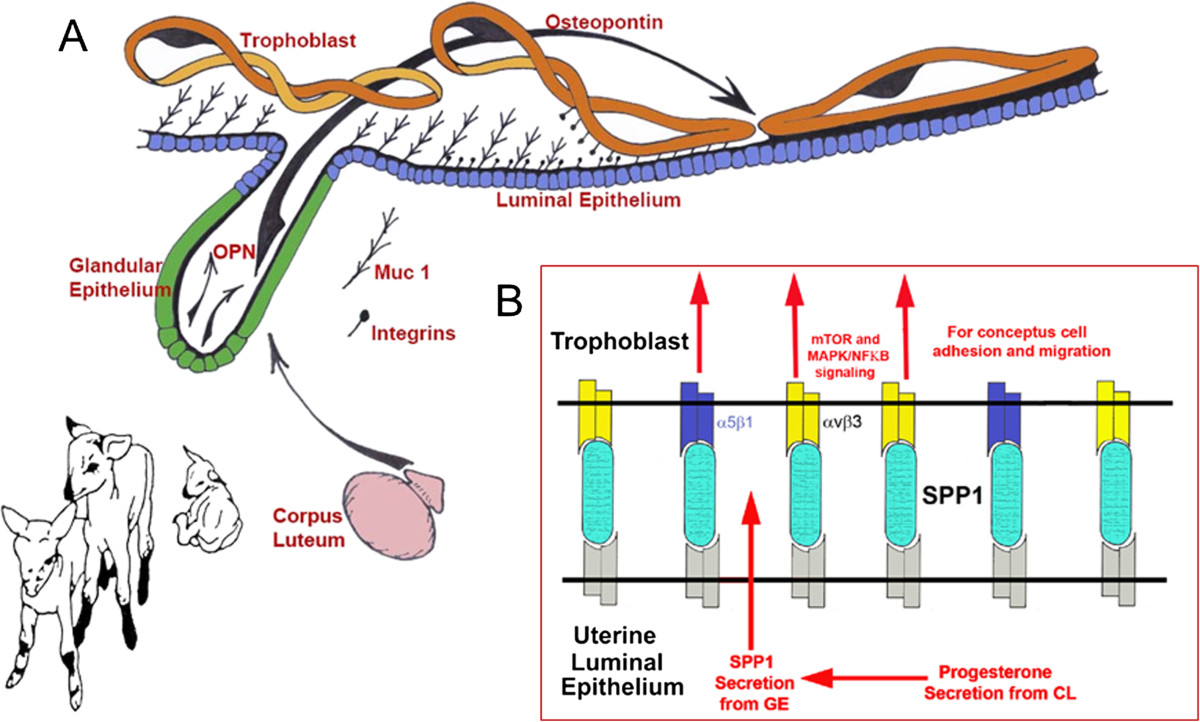
**Expression, regulation and proposed function of OPN produced by the uterine GE of pregnant sheep. A)** As the lifespan of the CL is extended as the result of the actions of interferon tau secretion from elongating ovine conceptuses (Trophoblast) they secrete progesterone. Progesterone then induces the synthesis and secretion of OPN (Osteopontin) from the uterine GE (Glandular Epithelium)[51]. The implantation cascade is initiated with down-regulation Muc 1 (the regulatory mechanism remains to be identified) on the LE surface to expose integrins on the LE and trophoblast surfaces for interaction with OPN to mediate adhesion of trophoblast to LE for implantation[29, 51, 52, 66]. **B)** *In vitro* experiments have identified the αvβ3 integrin receptor on trophoblast as a binding partner for OPN[66]. OPN then likely acts as a bridging ligand between αvβ3 on trophoblast and as yet unidentified integrin receptor(s) expressed on the opposing uterine LE. Note that the α5 integrin subunit was immunoprecipitated from membrane extracts of biotinylated oTr1 cells that were eluted from an OPN-Sepharose column, but the β1 integrin subunit, the only known binding partner for α5, could not be immunoprecipitated. Therefore, while we cannot definitively state that OPN binds α5β1 integrin on oTr1, we are reticent to exclude this possibility.

Affinity chromatography and immunoprecipitation experiments, similar to those described previously for pigs, determined whether αv, α4, α5, β1, β3, β5, and β6 integrins expressed by ovine trophectoderm cells (oTr1) directly bind OPN. Successful immunoprecipitation of labeled oTr1 integrins occurred with antibodies to αv and β3 integrin subunits, as well as an antibody to the integrin αvβ3 heterodimer. Antibody to the αv integrin subunit also precipitated a β chain, presumed to be the β3 integrin subunit, as an antibody to the β3 integrin subunit precipitated an α chain at the same relative size as the bands precipitated by an antibody to the αvβ3 heterodimer. Thus, the αvβ3 integrin on oTr1 cells binds OPN[66]. OPN binding to the αvβ3 integrin receptor induced *in vitro* focal adhesion assembly (see Figure 3), a prerequisite for adhesion and migration of trophectoderm, through activation of: 1) P70S6K via crosstalk between FRAP1/MTOR and MAPK pathways; 2) MTOR, PI3K, MAPK3/MAPK1 (Erk1/2) and MAPK14 (p38) signaling to stimulate trophectoderm cell migration; and 3) focal adhesion assembly and myosin II motor activity to induce migration of trophectoderm cells[66]. Collectively, results indicate that OPN binds αvβ3 integrin receptor to activate cell signaling pathways that act in concert to mediate adhesion, migration and cytoskeletal remodeling of trophectoderm cells essential for expansion and elongation of conceptuses and their attachment to uterine LE for implantation (Figure 4).

Focal adhesions, the hallmark of activated integrins, are prominent structures of cells grown in culture; however, they are rarely observed *in vivo*. It is noteworthy that large aggregations of focal adhesion-associated proteins that have been interpreted to be three dimensional focal adhesions are present at the uterine-placental interface of sheep[63]. By day 40 of pregnancy in sheep, the punctate apical surface staining of integrin receptor subunits identified in peri-implantation uterine LE and conceptus trophectoderm[52] is replaced by scattered large aggregates of αv, α4, β1, and β5 subunits in interplacentomal LE and trophectoderm/chorion cells. Integrin aggregates are observed only in gravid uterine horns of unilaterally pregnant sheep, demonstrating a requirement for trophectoderm attachment to LE, and aggregates increase in number and size through Day 120 of pregnancy[63]. Interestingly, no accumulation of β3 was observed even though ITGB3 is a prominent component of the uterine-placental interface during the peri-implantation period in sheep[52]. In some regions of the interplacentomal interface, greater subunit aggregation was seen on the uterine side, in other regions it was predominant on the placental side; whereas in some others, both uterine and placental epithelia exhibited prominent focal adhesions. However, by Day 120 of pregnancy, extensive focal adhesions were seen along most of the uterine-placental interface[63]. The placentomes, which provide hematotrophic support to the fetus and placenta, exhibited diffuse immunoreactivity for these integrins compared with interplacentomal regions perhaps due to extensive folding at this interplacentomal interface[63]. These results suggest that focal adhesion assembly at the uterine-placental interface reflects dynamic adaptation to increasing forces caused by the growing conceptus. Cooperative binding of multiple integrins to OPN deposited at the uterine-placental interface may form an adhesive mosaic to maintain a tight connection and increased tensile strength and signaling activity between uterine and placental surfaces along regions of epitheliochorial placentation in sheep.

Steady-state levels of OPN mRNA in total ovine endometrium remain constant between Days 20 and 40, increase 40-fold between Days 40 and 100, and remain maximal thereafter[18]. The major source of this OPN is uterine GE which undergoe hyperplasia through Day 50 followed by hypertrophy and maximal production of histotroph after Day 60[115]. Additionally, immunofluorescence microscopy demonstrated that the secreted 45-kDa OPN cleavage fragment is exclusively, continuously, and abundantly present along the apical surface of uterine LE, on trophectoderm, and along the entire uterine-placental interface of both interplacentomal and placentomal regions through Day 120 of the 147 day ovine pregnancy[115]. These findings definitively localize OPN as a secretory product of the GE to regions of intimate contact between conceptus and uterus, where OPN may influence fetal/placental development and growth, and mediate communication between placental and uterine tissues to support pregnancy to term.

Increases in OPN from GE are likely influenced by uterine exposure to progesterone, interferon-tau, and placental lactogen which constitute a “servomechanism” that activates and maintains endometrial remodeling, secretory function and uterine growth during gestation. Sequential treatment of ovariectomized ewes with progesterone, interferon tau, placental lactogen, and growth hormone results in GE development similar to that observed during normal pregnancy[126]. Administration of progesterone alone in these experiments induced expression of OPN in GE, and intrauterine infusion of interferon tau and placental lactogen to progesterone-treated ovariectomized ewes increased OPN mRNA levels above those for ewes treated with progesterone alone[126]. An attractive hypothesis for OPN expression in GE is that progesterone interacts with its receptor in GE to down-regulate the progesterone receptor. This removes a progesterone “block” to OPN synthesis, and subsequent increases of OPN expression by GE are augmented by stimulatory effects of placental lactogen. Current studies focus on defining the role(s) of OPN secreted from the uterine GE during later stages of pregnancy.

## Conclusions

Research conducted with pigs and sheep has significantly advanced understanding of the role(s) of OPN during implantation through exploitation of 1) the prolonged peri-implantation period of pregnancy when elongating conceptuses are free within the uterine lumen requiring extensive paracrine signaling between conceptus and endometrium, and 2) the protracted and incremental attachment cascade of trophectoderm to uterine LE during implantation. Although OPN is synthesized in different cell types (LE in pigs, GE in sheep) and is regulated by different hormones (conceptus estrogens in pigs, progesterone in sheep), nonetheless OPN protein localizes to the interface between the uterus and trophectoderm where it is well placed to serve as a bifunctional bridging ligand between integrins, expressed by uterine LE and conceptus trophectoderm, to mediate attachment for implantation. It is noteworthy that OPN has been reported to be a prominent component of the uterine-placental environment of other species including primates and rodents, and therefore knowledge gained about the physiology of OPN in sheep and pigs may have significant relevance to human pregnancy. Our understanding of events that underlie successful implantation and placentation across species has been and will likely continue to be advanced by studies of pigs and sheep as biomedical research models.

## Authors’ original submitted files for images

Below are the links to the authors’ original submitted files for images. Authors’ original file for figure 1 Authors’ original file for figure 2 Authors’ original file for figure 3 Authors’ original file for figure 4
