# Supplementary figures and images for: Osteopontin: a leading candidate adhesion molecule for implantation in pigs and sheep
Source: J Anim Sci Biotechnol. 2014 Dec 17;5:56. doi: 10.1186/2049-1891-5-56 (PMC4322467; doi:10.1186/2049-1891-5-56)

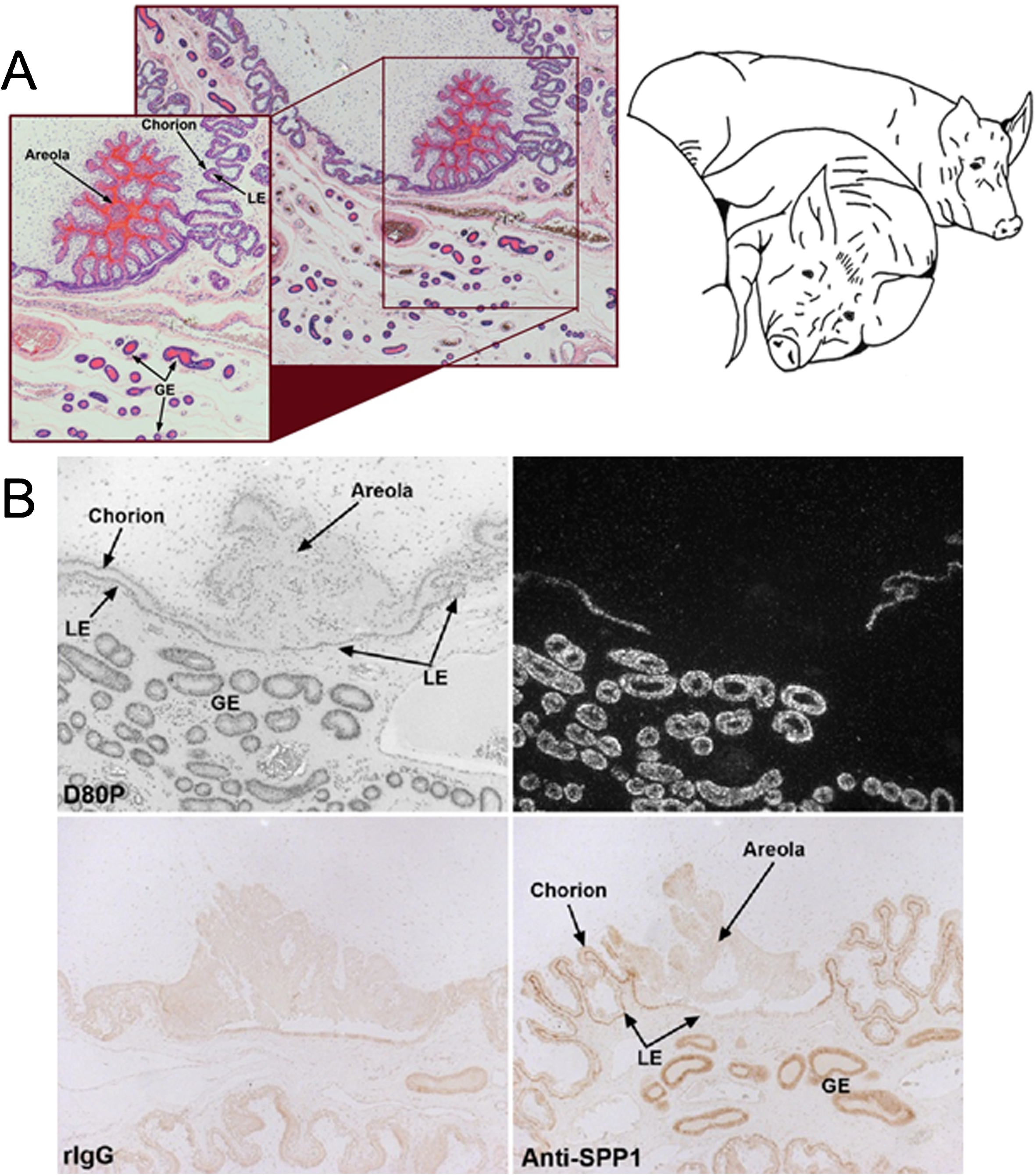

Supplement: Supplementary file 1 — Authors’ original file for figure 1 [file 40104_2014_131_MOESM1_ESM.tif]

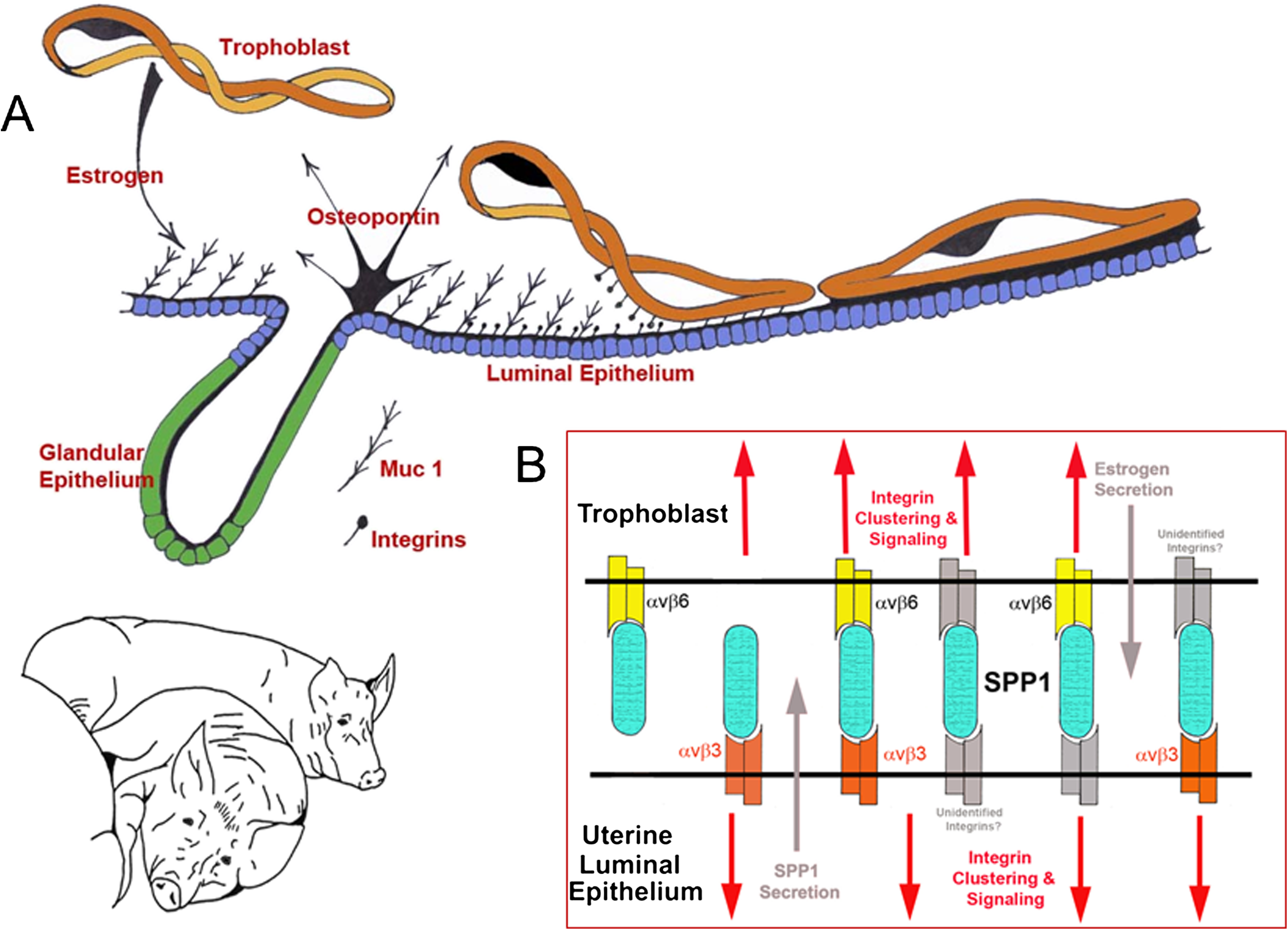

Supplement: Supplementary file 2 — Authors’ original file for figure 2 [file 40104_2014_131_MOESM2_ESM.tif]

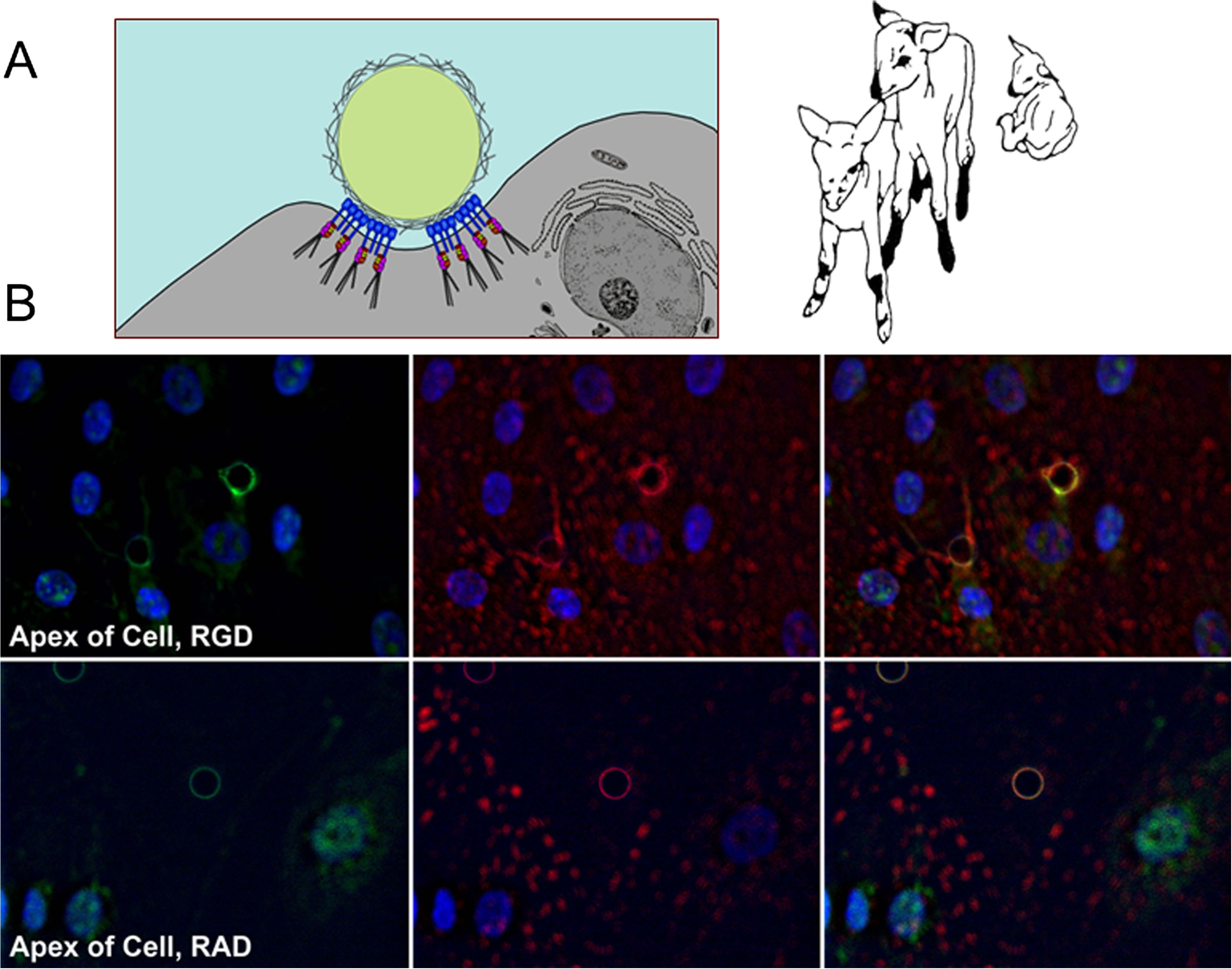

Supplement: Supplementary file 3 — Authors’ original file for figure 3 [file 40104_2014_131_MOESM3_ESM.tif]

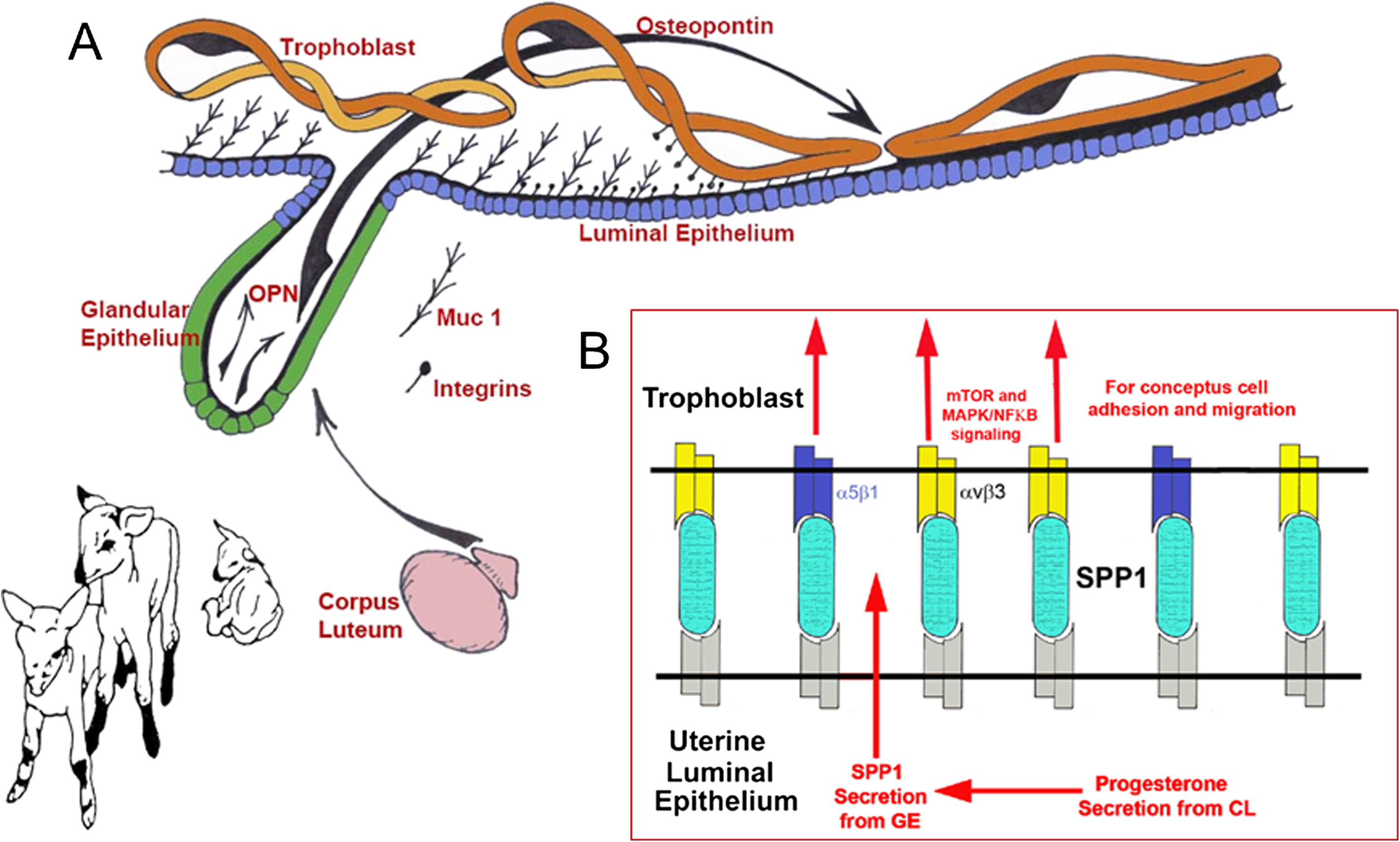

Supplement: Supplementary file 4 — Authors’ original file for figure 4 [file 40104_2014_131_MOESM4_ESM.tif]
